# Combining tau-PET and fMRI meta-analyses for patient-centered prediction of cognitive decline in Alzheimer’s disease

**DOI:** 10.1186/s13195-022-01105-5

**Published:** 2022-11-07

**Authors:** Davina Biel, Ying Luan, Matthias Brendel, Paul Hager, Anna Dewenter, Alexis Moscoso, Diana Otero Svaldi, Ixavier A. Higgins, Michael Pontecorvo, Sebastian Römer, Anna Steward, Anna Rubinski, Lukai Zheng, Michael Schöll, Sergey Shcherbinin, Michael Ewers, Nicolai Franzmeier, the Alzheimer’s Disease Neuroimaging Initiative

**Affiliations:** 1 Institute for Stroke and Dementia Research (ISD), University Hospital, LMU Munich, 81377 Munich, Germany; 2 Department of Nuclear Medicine, University Hospital, LMU Munich, Munich, Germany; 3 Wallenberg Centre for Molecular and Translational Medicine, University of Gothenburg, Gothenburg, Sweden; 4 Department of Psychiatry and Neurochemistry, Institute of Neuroscience and Physiology, The Sahlgrenska Academy, University of Gothenburg, Gothenburg, Sweden; 5 Eli Lilly and Company, Indianapolis, IN USA; 6 Avid Radiopharmaceuticals, Philadelphia, PA USA; 7 Department of Neurology, University Hospital, LMU Munich, Munich, Germany; 8 Department of Neurodegenerative Disease, UCL Institute of Neurology, London, UK; 9 German Center for Neurodegenerative Diseases (DZNE), Munich, Germany; 10 Munich Cluster for Systems Neurology (SyNergy), Munich, Germany

**Keywords:** Alzheimer’s disease, Tau-PET, fMRI, Cognitive decline, Precision medicine

## Abstract

**Background:**

Tau-PET is a prognostic marker for cognitive decline in Alzheimer’s disease, and the heterogeneity of tau-PET patterns matches cognitive symptom heterogeneity. Thus, tau-PET may allow precision-medicine prediction of individual tau-related cognitive trajectories, which can be important for determining patient-specific cognitive endpoints in clinical trials. Here, we aimed to examine whether tau-PET in cognitive-domain-specific brain regions, identified via fMRI meta-analyses, allows the prediction of domain-specific cognitive decline. Further, we aimed to determine whether tau-PET-informed personalized cognitive composites capture patient-specific cognitive trajectories more sensitively than conventional cognitive measures.

**Methods:**

We included Alzheimer’s Disease Neuroimaging Initiative (ADNI) participants classified as controls (i.e., amyloid-negative, cognitively normal, *n* = 121) or Alzheimer’s disease-spectrum (i.e., amyloid-positive, cognitively normal to dementia, *n* = 140), plus 111 AVID-1451-A05 participants for independent validation (controls/Alzheimer’s disease-spectrum=46/65). All participants underwent baseline ^18^F-flortaucipir tau-PET, amyloid-PET, and longitudinal cognitive testing to assess annual cognitive changes (i.e., episodic memory, language, executive functioning, visuospatial). Cognitive changes were calculated using linear mixed models. Independent meta-analytical task-fMRI activation maps for each included cognitive domain were obtained from the Neurosynth database and applied to tau-PET to determine tau-PET signal in cognitive-domain-specific brain regions. In bootstrapped linear regression, we assessed the strength of the relationship (i.e., partial *R*^2^) between cognitive-domain-specific tau-PET vs. global or temporal-lobe tau-PET and cognitive changes. Further, we used tau-PET-based prediction of domain-specific decline to compose personalized cognitive composites that were tailored to capture patient-specific cognitive decline.

**Results:**

In both amyloid-positive cohorts (ADNI [age = 75.99±7.69] and A05 [age = 74.03±9.03]), cognitive-domain-specific tau-PET outperformed global and temporal-lobe tau-PET for predicting future cognitive decline in episodic memory, language, executive functioning, and visuospatial abilities. Further, a tau-PET-informed personalized cognitive composite across cognitive domains enhanced the sensitivity to assess cognitive decline in amyloid-positive subjects, yielding lower sample sizes required for detecting simulated intervention effects compared to conventional cognitive endpoints (i.e., memory composite, global cognitive composite). However, the latter effect was less strong in A05 compared to the ADNI cohort.

**Conclusion:**

Combining tau-PET with task-fMRI-derived maps of major cognitive domains facilitates the prediction of domain-specific cognitive decline. This approach may help to increase the sensitivity to detect Alzheimer’s disease-related cognitive decline and to determine personalized cognitive endpoints in clinical trials.

**Supplementary Information:**

The online version contains supplementary material available at 10.1186/s13195-022-01105-5.

## Background

Alzheimer’s disease (AD) is characterized by the accumulation of beta-amyloid (Aβ) and tau pathology ensuing neurodegeneration and cognitive decline [1, 2]. While neuroimaging and fluid biomarkers of Aβ and tau are routinely established for AD diagnosis [2, 3], reliable prognosis of cognitive decline remains a critical challenge, which is further complicated by the considerable heterogeneity in symptom manifestation and disease progression [4–6].

Accurate prediction of cognitive decline in AD will be, however, particularly important for clinical trials to (i) stratify patients by progression risk and/or (ii) determine patient-specific clinical endpoints that allow sensitive assessment of heterogeneous cognitive trajectories within trial-typical 1–2-year follow-ups [4, 7, 8]. Elevated tau-PET is closely associated with the development of cognitive deficits in AD, and recent studies also support tau-PET as a promising prognostic marker for cognitive decline, outperforming the prognostic accuracy of amyloid-PET and MRI-assessed neurodegeneration [9–11]. Specifically, elevated tau-PET has been associated with faster global cognitive decline and clinical conversion to mild cognitive impairment (MCI) and AD dementia [9, 10, 12, 13]. Moreover, cross-sectional studies reported that tau-PET patterns closely mirror clinical AD heterogeneity, including occipital tau-PET in posterior cortical atrophy (i.e., visual-variant AD), left-hemispheric tau-PET in language-variant AD, and mesial-temporal-lobe tau in amnestic AD [14, 15]. This suggests a strong clinico-pathological correspondence between symptom manifestation and tau-PET patterns. Thus, mapping tau-PET to brain networks associated with cognitive domains typically affected in AD may allow the prediction of cognitive-domain-specific decline, to facilitate the assessment of patient-specific cognitive trajectories in clinical trials targeting tau pathology [16].

To test this, we obtained data from two independent cohorts from the Alzheimer’s disease neuroimaging initiative (ADNI, *N* = 261) and the A05 cohort (*N* = 111) covering healthy controls (i.e., cognitively normal Aβ−) and AD-spectrum patients (i.e., Aβ+) with baseline flortaucipir tau-PET and amyloid-PET. Further, all participants were characterized on different cognitive domains including episodic memory (MEM), language (LAN), executive functioning (EF), and visuospatial abilities (VS) across ~1.5–2 years of follow-up, which matches follow-up periods of several recent phase 2b&3 clinical trials (e.g., EMERGE/ENGAGE) [8]. To assess the association between tau-PET and cognitive-domain-specific decline, we obtained meta-analytical maps of task-fMRI studies from Neurosynth [17, 18] to determine brain regions that are consistently associated with MEM/LAN/EF/VS across ~1900 task-fMRI studies. We then mapped patient-specific tau-PET to cognitive-domain-specific brain activation maps and tested whether determining baseline tau-PET in regions involved in a given cognitive domain improves the prediction of cognitive-domain-specific decline compared to conventional tau-PET metrics (i.e., global/temporal-lobe tau-PET standardized uptake value ratios [SUVRs]) that have been suggested as prognostic markers in AD [9, 10]. Lastly, we used tau-PET predicted domain-specific decline to compute patient-specific weighted cognitive composites, which we hypothesized to capture individual tau-related cognitive trajectories more sensitively than commonly used cognitive outcomes. Using simulated interventions, we then tested whether tau-PET-informed cognitive composites (i.e., personalized endpoints) [19] improve the sensitivity to detect treatment effects compared to conventional cognitive scores.

## Materials and methods

### Participants

#### ADNI

We included 261 ADNI participants (inclusion criteria: https://adni.loni.usc.edu/wp-content/uploads/2010/09/ADNI_GeneralProceduresManual.pdf) based on the availability of ^18^F-florbetapir/^18^F-florbetaben amyloid-PET, ^18^F-flortaucipir tau-PET, longitudinal cognitive assessments (>1 follow-up), demographics (age, sex, education), clinical status, and APOE genotyping. All baseline data were obtained within 6 months; clinical status was classified by ADNI investigators as cognitively normal (CN, Mini-Mental State Examination [MMSE] ≥ 24, Clinical Dementia Rating [CDR] = 0, non-depressed), MCI (MMSE ≥ 24, CDR = 0.5, objective memory-impairment on education-adjusted Wechsler Memory Scale II, preserved activities of daily living), or demented (MMSE = 20–26, CDR ≥ 0.5, NINCDS/ADRDA criteria for probable AD). Subjects with non-AD-related cognitive impairment (i.e., Aβ− MCI/dementia) were excluded. Subjects were classified as APOE4 risk allele carriers when at least one ε4 allele was detected. Ethics approval was obtained by ADNI investigators; all study participants provided written informed consent.

#### A05

A total of 111 participants were selected from the AVID-1451-A05 phase 2/3 trial (NCT02016560; inclusion criteria: https://clinicaltrials.gov/ProvidedDocs/60/NCT02016560/Prot_000.pdf), based on the availability of ^18^F-florbetapir amyloid-PET, ^18^F-flortaucipir tau-PET, longitudinal cognitive assessments (≥ 1follow-up), demographics (age, sex), clinical status, and APOE genotyping. Continuous measures on years of education were not available for all participants and thus not included. All baseline data were obtained within 30 days. Participants were classified as CN (MMSE ≥ 29, no history of cognitive impairment), MCI (24 ≤ MMSE < 29, showing MCI according to NIA-AA working group’s diagnostic guidelines) [20], and AD dementia (10 < MMSE < 24, showing possible or probable AD based on NIA-AA working group’s diagnostic guidelines) [21]. As for ADNI, subjects with non-AD-related cognitive impairment (i.e., Aβ− MCI/dementia) were excluded. Subjects were classified as APOE4 risk allele carriers when at least one ε4 allele was detected. The study was approved by the clinical investigator’s local Institutional Review Board; all participants provided written informed consent.

### fMRI meta-analyses of cognitive-domain-specific brain activation and tau-PET assessments

**Fig. 1:**
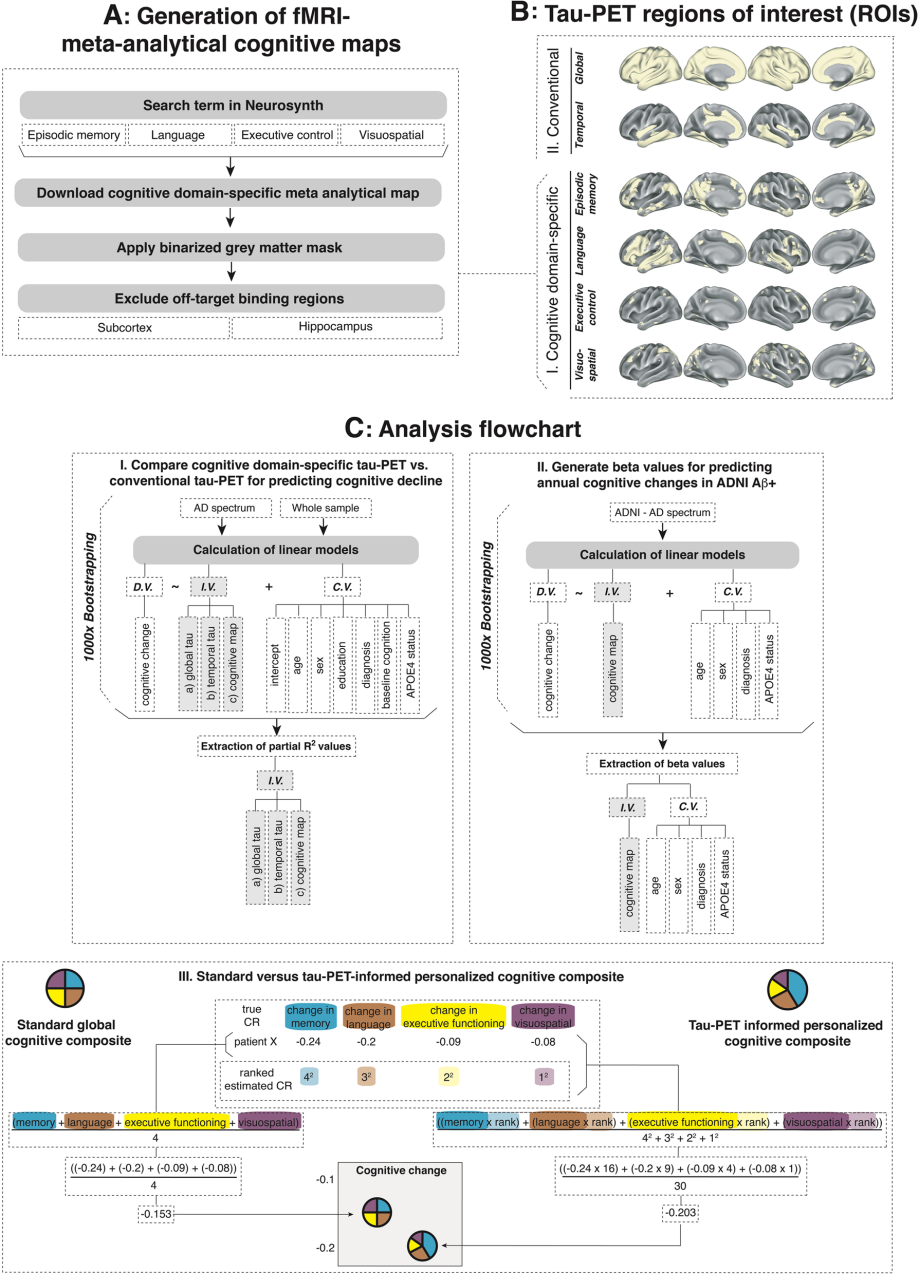
Generation of meta-analytical task-fMRI maps for assessing cognitive-domain-specific tau-PET (**A**), surface rendering of regions of interests (ROIs) applied to tau-PET (**B**), and analysis flowchart (**C**). First, we compared the predictive accuracy of global tau-PET, temporal tau-PET, and cognitive-domain-specific tau-PET for predicting future cognitive decline using bootstrapped linear regressions. Note that separate regression models were run for each tau-PET ROI *(CI)*. Second, we ran 1000 bootstrapped linear regression models in ADNI Aβ+ and extracted beta values for each variable of the regression model (*CII*), which were later used for assessing patient-specific cognitive composites in both ADNI Aβ+ and A05 Aβ+ (*CIII*). Specifically, we applied the 1000 bootstrapped linear model equations to subject level data to determine predicted cognitive change rates for each Aβ+ subject of the ADNI and A05 sample. Based on the rank of the predicted cognitive change rates, we then determined a tau-PET-informed personalized cognitive composite. Abbreviations: DV, dependent variable; IV, independent variable; CV, control variable; CR, change rate

To determine tau-PET in regions that are critical for a given cognitive domain (i.e., MEM/LAN/EF/VS), we obtained meta-analytical brain activation maps from task-based fMRI studies from Neurosynth (https://neurosynth.org/analyses/terms/, see Additional file 1: Methods) using the search terms: *episodic memory* (included studies, *N* = 332), *language* (included studies, *N* = 1101), *executive control* (included studies, *N* = 230), and *visuospatial* (included studies, *N* = 267). Methodological details on Neurosynth-based meta-analyses have been described previously (see https://neurosynth.org/faq/) [17]. Cognitive-domain-specific meta-analytical task-fMRI maps were binarized and masked with cortical gray matter to exclude typical tau-PET off-target binding regions (i.e., hippocampus/subcortex) [22, 23] and applied to spatially normalized SUVR-transformed tau-PET images to extract tau-PET signal within cognitive-domain-specific brain regions (for details on PET acquisition and preprocessing see Additional file 1: Methods). To further minimize influences of flortaucipir off-target binding, we used voxel-wise two-component Gaussian-mixture-modeling to transform SUVRs to tau-positivity probabilities (TPP) [24, 25]. Repeating this procedure for each task-fMRI map yielded cognitive-domain-specific tau-PET signals for MEM/LAN/EF/VS (Fig. 1A, B (I)). To compare the accuracy of cognitive-domain-specific tau-PET for predicting cognitive trajectories against conventional tau-PET measures, we obtained global and temporal-lobe tau-PET SUVRs, which have been previously shown to capture AD-related tau accumulation and predict cognitive decline [9, 10, 26, 27]. For global tau-PET, we determined average neocortical tau-PET, while excluding typical off-target binding regions (hippocampus, subcortex, cerebellum [22, 23]; Fig. 1B (II)). The temporal-lobe ROI included Braak-stage ROIs 1, 3, and 4 (Fig. 1B (II)) [24, 28].

### Assessment of cognitive changes

For both samples, we obtained composites of MEM/LAN/EF/VS to assess cognitive-domain-specific changes. In ADNI, we used pre-established composites including ADNI-MEM [29], ADNI-LAN, ADNI-EF, and ADNI-VS (https://adni.bitbucket.io/reference/docs/UWNPSYCHSUM/adni_uwnpsychsum_doc_20200326.pdf) [30]. In A05, we generated composites (i.e., A05-MEM, A05-LAN, A05-EF, A05-VS) based on available cognitive tests using a pre-established approach (see Additional file 1: Methods, Table S1) [31, 32]. For each cognitive composite and cohort, annual cognitive changes were determined by fitting linear mixed models with cognitive composites as the dependent variable and time (i.e., years from baseline) as the independent variable, with subject-specific random slope and intercept [10, 33]. Thereby, we derived subject-specific annual cognitive change rates for each cognitive domain.

### Statistical analysis

All analyses were computed using R statistical software version 4.0.2 (https://www.R-project.org/).

Differences in baseline characteristics between diagnostic groups were tested using ANOVAs for continuous and chi-squared tests for categorical data.

To compare the accuracy of different tau-PET ROIs (i.e., cognitive-domain-specific/global/temporal, Fig. 1B) for predicting cognitive decline, we performed bootstrapped linear regression with 1000 iterations per cognitive domain and tau-PET ROI. Within each iteration, cognitive changes on MEM/LAN/EF/VS were included as dependent variables, and tau-PET ROIs (i.e., cognitive-domain-specific/global/temporal) as independent variables. For ADNI, all models were controlled for age, sex, education, clinical status, APOE4 status, and baseline performance of the respective cognitive composite. For A05, continuous measures of education were not consistently available; hence, models were corrected for age, sex, clinical status, APOE4 status, and baseline performance of the respective cognitive composite. In each iteration, resulting partial *R*^2^ values (variance explained in cognitive change) of the respective tau-PET ROI were extracted. The resulting partial *R*^2^ distributions of the different ROIs were then compared using paired t-tests and standardized differences were calculated using Cohen’s *d*. In addition, 95% confidence intervals (CI) of bootstrapped partial *R*^2^ distributions were determined for non-parametric comparisons. Main analyses were conducted in AD-spectrum patients (i.e., Aβ+) and exploratorily repeated in the pooled CN Aβ− plus AD-spectrum sample (Fig. 1C (I)).

Next, we aimed to determine patient-specific cognitive composites that are informed by the baseline tau-PET-based prediction of cognitive decline, using ADNI as a discovery sample and A05 as a validation sample. Within ADNI Aβ+, we ran 1000 bootstrapped regressions controlled for age, sex, clinical status, and APOE4 status to extract 1000 beta-values of the association between cognitive-domain-specific tau-PET and cognitive changes (i.e., ADNI-MEM/ADNI-LAN/ADNI-EF/ADNI-VS) for each variable of the regression model (Fig. 1C (II)). Note, that the cognitive composites which were used for building the model (e.g., ADNI-MEM) included slightly differed tests than the cognitive composites of the validation cohort (e.g., A05-MEM), ensuring additional generalizability of the model and independence from specific tests for MEM/LAN/EF/VS.

Next, we entered patient-specific baseline data of both ADNI (i.e., discovery) and A05 (i.e., validation) subjects in the 1000 linear model equations to determine mean patient-specific estimates of cognitive decline. To generate a personalized cognitive composite, we computed squared ranks of predicted cognitive changes across MEM/LAN/EF/VS for each subject (i.e., from 1^2^=slowest predicted decline up to 4^2^=fastest predicted decline), in order to maximize the information weight of cognitive domains with fast predicted cognitive decline. For each subject, cognitive changes (see the “Assessment of cognitive changes” section) were then weighted by the tau-PET informed squared rank (i.e., 1^2^, 2^2^, 3^2^, 4^2^; see Fig. 1C (III)) to determine a weighted, personalized cognitive composite that summarizes cognitive changes based on the degree of tau-PET-predicted cognitive decline. Using a paired *t*-test, we then tested whether tau-PET-informed personalized cognitive composites show stronger cognitive changes compared to conventional cognitive composites that average all cognitive domains.

Lastly, we exploratorily tested whether tau-PET-informed personalized cognitive composites are more sensitive for detecting treatment effects compared to conventional measures of global cognition and memory. To that end, we simulated interventions by attenuating cognitive changes by 20/30/40% and calculated required sample sizes for detecting simulated intervention effects using the R-package *pwr* (settings: two-sample *t*-test, comparing actual vs. attenuated cognitive changes; two-tailed, alpha = 0.05, power = 0.8; see https://cran.r-project.org/web/packages/pwr/pwr.pdf). This analysis was performed for tau-PET-informed personalized cognitive composites, an unweighted cognitive composite (i.e., *z*-score mean across actual change rates of ADNI/A05-MEM/LAN/EF/VS), as well as actual change rates of MEM (ADNI-MEM/A05-MEM) [34]. The latter one was selected, since episodic memory decline is one of the hallmark cognitive symptoms in AD as extensively reported before [35–37]. The analyses were repeated in the ADNI and A05 AD spectrum groups excluding APOE4 status as a covariate to test whether our models also apply in more simple settings without APOE genotype availability (see Additional file 1 [ADNI: *n* = 149; A05: *n* = 67]).

## Results

### Sample characteristics

We included 183/53/25 CN/MCI/demented ADNI subjects (age = 74.89±7.48 years) and 51/35/25 CN/MCI/demented A05 individuals (age = 71.29±10.03 years). Descriptive statistics stratified by cohort, clinical status, and amyloid status are shown in Table 1. Group-mean tau-PET SUVRs are shown in Additional file 1: Fig. S1.

**Table 1:** Subjects characteristics

**ADNI (*N*** **= 261)**	**CN Aβ−** **(*n*** **= 121)**	**CN Aβ+** **(*n*** **= 62)**	**MCI Aβ+** **(*n*** **= 53)**	**Dementia Aβ+** **(*n*** **= 25)**	***p*-value**
Age in years	73.61 (7.05)	75.85 (7.08)	75.87 (7.13)	76.60 (10.21)	0.079
Sex (male/female)	52/69	27/35	31/22	13/12	0.247
APOE4 (non-carriers/carriers)	88/33	28/34	22/31	9/16	<0.001
Years of education	16.69 (2.46)	16.87 (2.28)	15.89 (2.80)	15.84 (2.49)	0.075
MEM change rate	0.045 (0.064)^c,d^	0.026 (0.071)^c,d^	−0.084 (0.075)^a,b,d^	−0.179 (0.081)^a,b,c^	<0.001
LAN change rate	-0.044 (0.028)^c,d^	−0.047 (0.030)^c,d^	−0.084 (0.037)^a,b,d^	−0.132 (0.055)^a,b,c^	<0.001
EF change rate	-0.065 (0.035)^b,c,d^	−0.086 (0.034)^a,d^	−0.095 (0.031)^a^	−0.110 (0.028)^a,b^	<0.001
VS change rate	−0.004 (0.007)^c,d^	−0.006 (0.008)^d^	−0.009 (0.010)^a,d^	−0.022 (0.015)^a,b,c^	<0.001
Global tau-PET SUVR	1.06 (0.07)^c,d^	1.11 (0.11)^c,d^	1.21 (0.22)^a,b,d^	1.46 (0.46)^a,b,c^	<0.001
Temporal-lobe tau-PET SUVR	1.13 (0.09)^c,d^	1.22 (0.15)^c,d^	1.39 (0.29)^a,b,d^	1.71 (0.47)^a,b,c^	<0.001
Mean cognitive follow-up in years	2.02 (0.80)^b,c,d^	1.64 (0.69)^a^	1.51 (0.72)^a^	1.48 (0.70)^a^	<0.001
Mean cognitive follow-up visits	2.33 (0.55)	2.32 (0.54)	2.36 (0.62)	2.32 (0.63)	0.986
**A05 (*N*** **= 111)**	**CN Aβ−** **(*n*** **= 46)**	**CN Aβ+** **(*n*** **= 5)**	**MCI Aβ+** **(*n*** **= 35)**	**Dementia Aβ+** **(*n*** **= 25)**	***p*-value**
Age in years	67.41 (10.19)^d^	77.8 (7.01)	72.06 (8.77)	76.04 (9.34)^a^	0.001
Sex (male/female)	26/20	3/2	19/16	11/14	0.762
APOE4 (non-carriers/carriers)	37/9	3/2	16/17	7/18	<0.001
MEM change rate	0.022 (0.027)^c,d^	0.015 (0.032)^b,d^	−0.019 (0.026)^a,b^	−0.038 (0.011)^a,b^	<0.001
LAN change rate	−0.001 (0.031)^c,d^	0.009 (0.018)^d^	−0.031 (0.030)^a,d^	−0.070 (0.049)^a,b,c^	<0.001
EF change rate	0.006 (0.026)^c,d^	0.009 (0.010)^c,d^	−0.040 (0.040)^a,b,d^	−0.077 (0.048)^a,b,c^	<0.001
VS change rate	0.005 (0.027)^c,d^	0.0007 (0.021)^d^	−0.032 (0.054)^a,d^	−0.107 (0.097)^a,b,c^	<0.001
Global tau-PET SUVR	1.01 (0.08)^c,d^	1.00 (0.09)^d^	1.22 (0.29)^a^	1.36 (0.32)^a,b^	<0.001
Temporal-lobe tau-PET SUVR	1.08 (0.10)^c,d^	1.08 (0.09)^d^	1.37 (0.30)^a^	1.52 (0.31)^a,b^	<0.001
Mean cognitive follow-up in years	1.47 (0.15)	1.5 (0)	1.33 (0.32)	1.38 (0.28)	0.067
Mean cognitive follow-up visits	2.96 (0.21)	3 (0)	2.77 (0.43)	2.84 (0.37)	0.067

In ADNI CN: MMSE ≥ 24, CDR = 0, non-depressed; MCI: MMSE ≥ 24, CDR = 0.5, objective memory-impairment on education-adjusted Wechsler Memory Scale II, preserved activities of daily living; demented: MMSE = 20–26, CDR ≥ 0.5, NINCDS/ADRDA criteria for probable AD

In A05 CN: MMSE ≥ 29, no history of cognitive impairment; MCI: 24 ≤ MMSE < 29, showing MCI according to NIA-AA working group’s diagnostic guidelines; demented: 10 < MMSE < 24, showing possible or probable AD based on NIA-AA working group’s diagnostic guidelines

Values are presented as mean (SD); *p*-values were derived from ANOVAs for continuous measures and from chi-squared tests for categorical measures

*MEM*, episodic memory composite score; *LAN*, language composite score; *EF*, executive functioning composite score; *VS*, visuospatial composite score

Mean values significantly (*p* < 0.05, post-hoc tests) different from:

^a^CN Aβ**−**

^b^CN Aβ+

^c^MCI Aβ+

^d^Dementia Aβ+

### Cognitive-domain-specific tau-PET outperforms conventional tau-PET metrics for predicting domain-specific cognitive decline

**Fig. 2:**
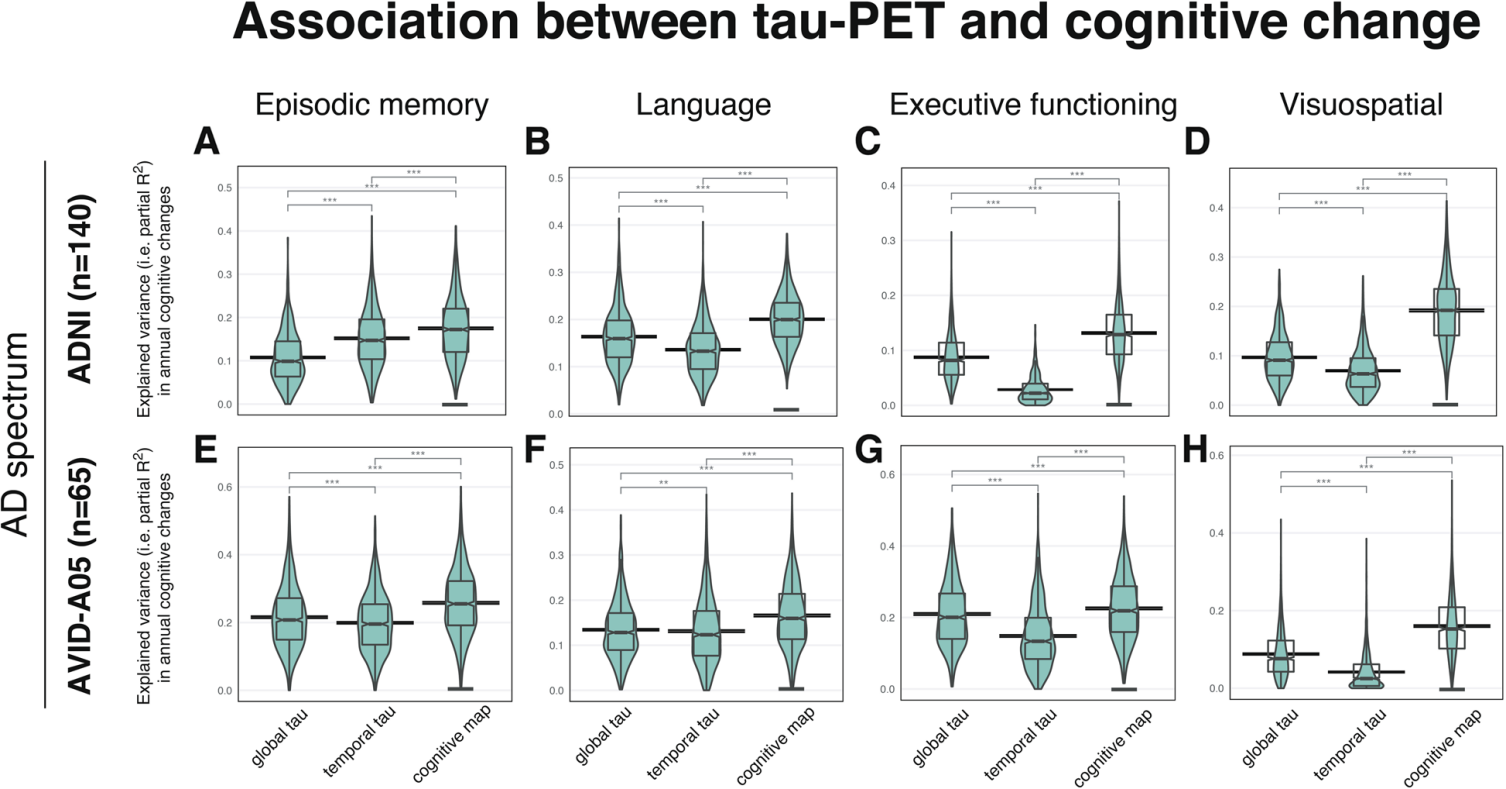
Bootstrapped linear models (1000 iterations) revealed that partial *R*^2^ values (explained variance in annual cognitive changes) were higher for cognitive-domain-specific tau than for global or temporal tau-PET. The boxplots illustrate the partial *R*^2^ distributions for episodic memory, language, executive functioning, and visuospatial abilities within the AD-spectrum (ADNI cohort: **A**–**D**; A05 cohort: **E**–**H**). The models were controlled for age, sex [in ADNI: education], clinical status, baseline score of the respective cognitive test, and APOE4 status. *R*^2^-distributions across different regions of interests (global vs. temporal vs. cognitive-domain-specific tau-PET) were compared with paired t-tests. 95% confidence intervals for the mean are displayed as horizontal lines. Within each panel, the tau-PET ROI with the highest explained variance is highlighted by an underscore. *p < 0.05, **p < 0.01, ***p < 0.001

First, we determined the accuracy of cognitive-domain-specific tau-PET vs. global and temporal-lobe tau-PET for predicting future decline in MEM/LAN/EF/VS. In ADNI Aβ+, bootstrapped linear regression revealed cognitive-domain-specific tau-PET as a better predictor of cognitive decline than global/temporal tau-PET for MEM (cognitive-domain-specific/global/temporal tau-PET: partial *R*^2^ = 0.175/0.108/0.152; Fig. 2A), LAN (cognitive-domain-specific/global/temporal tau-PET: partial *R*^2^ = 0.201/0.164/0.136; Fig. 2B), EF (cognitive-domain-specific/global/temporal tau-PET: partial *R*^2^ = 0.132/0.088/0.029; Fig. 2C), and VS (cognitive-domain-specific/global/temporal tau-PET: partial *R*^2^ = 0.192/0.097/0.070; Fig. 2D). Further, 95%CIs did not overlap between bootstrapped partial *R*^2^-distributions of the cognitive-domain-specific tau-PET and global/temporal tau-PET ROIs, providing non-parametric support that cognitive-domain-specific tau-PET explains more variance in cognitive decline than conventional tau-PET measures (Fig. 2A–D). Congruent results were obtained for the A05 Aβ+ validation cohort for all cognitive tests (Fig. 2E–H). Detailed results of bootstrapped regressions are provided in Additional file 1: Tables S2&3. When repeating these analyses in the pooled CN Aβ− plus Aβ+ sample, we obtained largely congruent results in both cohorts except for LAN in A05 (Additional file 1: Table S3). Using SUVRs rather than Gaussian mixture model transformed tau-PET signal of the cognitive-domain-specific ROI yielded consistent results except for ADNI-MEM and ADNI/A05-LAN (see Additional file 1: Table S4&5). The results remained consistent when excluding APOE4 status as covariate from the model (Additional file 1: Table S6-10 and Figure S2). Together, these findings suggest that combining tau-PET with meta-analytical task-fMRI maps of major cognitive functions improves the prediction of domain-specific cognitive decline compared to conventional global/temporal-lobe tau-PET measures in AD.

### A tau-PET-informed cognitive composite increases the sensitivity to assess cognitive decline

**Fig. 3:**
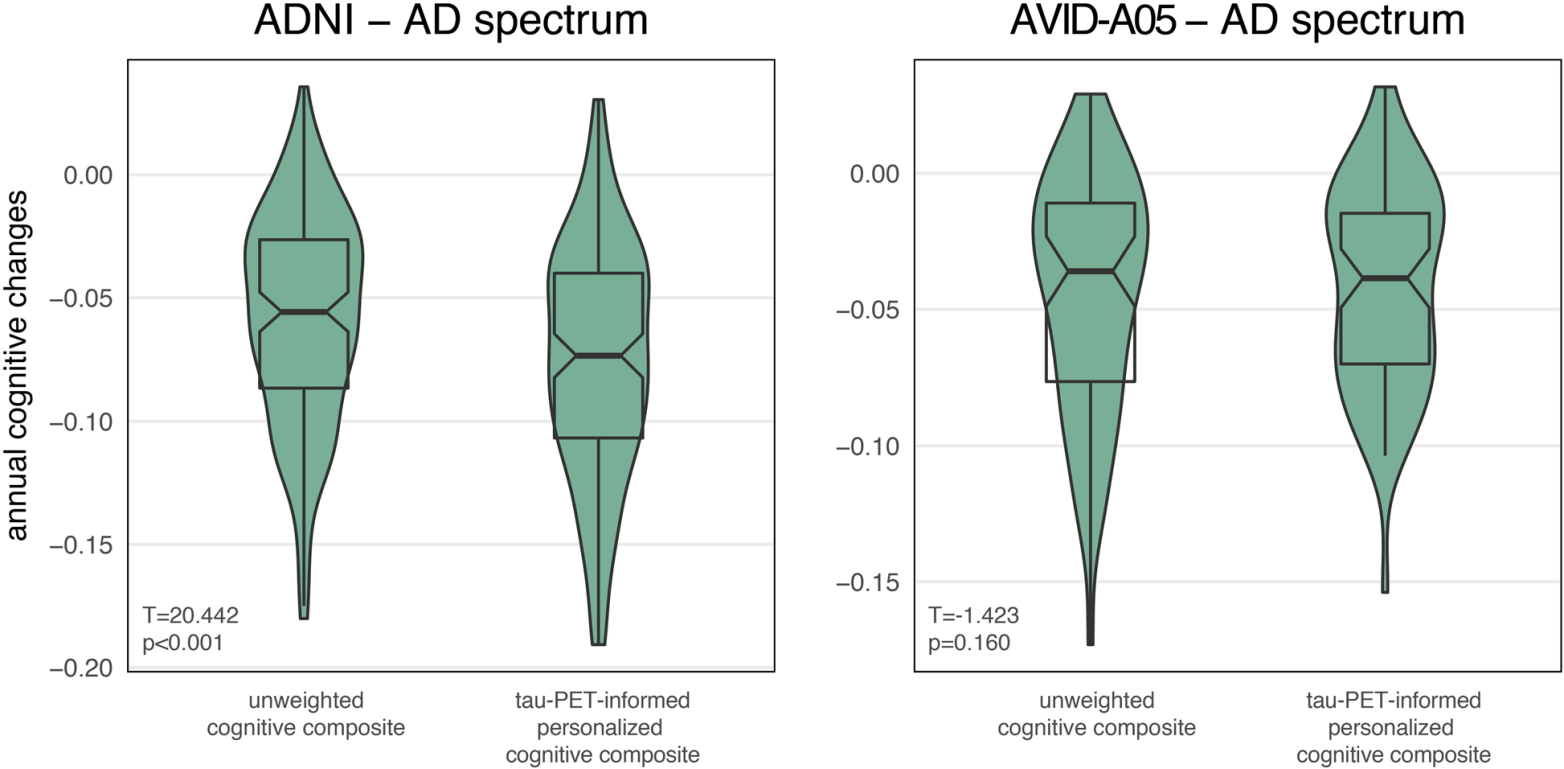
Comparison of unweighted (i.e., average of MEM/LAN/EF/VS) and tau-PET-informed personalized cognitive composites for AD-spectrum patients (i.e., Aβ+) within the ADNI cohort (**A**) and the A05 cohort (**B**). Statistics were derived from paired *t*-tests. The model for the tau-PET-informed personalized cognitive composites was controlled for age, sex, clinical status, and APOE4 status

Next, we used tau-PET-based prediction of cognitive decline to determine personalized cognitive composites to better capture individual tau-related cognitive trajectories. Specifically, we ran 1000 bootstrapped regression models in ADNI Aβ+ (i.e., discovery sample) to determine beta-estimates for the association between cognitive-domain-specific tau-PET and cognitive decline, controlling for age, sex, clinical status, and APOE4 status. To derive patient-specific estimates for cognitive decline in ADNI Aβ+, we then entered individual tau-PET measures and covariates in bootstrapped linear model equations to determine mean estimates for cognitive decline. For each subject, we then square-ranked the resulting cognitive change rates on the four cognitive domains (i.e., from 1^2^=slowest predicted cognitive decline up to 4^2^=fastest predicted cognitive decline), to determine a rank-weighted cognitive composite that maximizes the information-weight of those cognitive domains in which cognitive decline is expected based on baseline tau-PET. The same linear model equations derived from ADNI Aβ+ were then applied to A05 Aβ+ for validation. In addition, we determined an “unweighted” cognitive composite as the average *z*-score across all four cognitive domains. We found that the tau-PET-informed patient-specific cognitive composite showed faster longitudinal decline than the unweighted cognitive composite in ADNI Aβ+ (*T* = 20.442, *p* < 0.001, Cohen’s *d*_paired_ = 1.728) but not in A05 Aβ+ (*T* = −1.423, *p* = 0.160, Cohen’s *d*_paired_ = 0.176; Fig. 3). When repeating the analysis excluding APOE4 status as covariate, we found a faster longitudinal decline of the tau-PET-informed patient-specific cognitive composite in ADNI (*T* = 15.624, *p* < 0.001, Cohen’s *d*_paired_ = 1.280) and in A05 (*T* = 2.758, *p* = 0.008, Cohen’s *d*_paired_ = 0.337; Additional file 1: Figure S3).

### Patient-centered cognitive composites can reduce the sample sizes to detect treatment effects in clinical trials

Lastly, we exploratorily assessed whether using tau-PET-informed personalized cognitive composites as an endpoint in clinical trials increases the sensitivity to detect intervention effects. To this end, we compared estimated sample sizes for simulated intervention effects (i.e., 20/30/40% attenuated cognitive decline) for different cognitive endpoints in Aβ+. Specifically, we calculated estimated sample sizes for MEM (ADNI-MEM/A05-MEM), as well as unweighted (i.e., mean across ADNI/A05-MEM/LAN/VIS/EF) and tau-PET-informed personalized cognitive composites. Across both the ADNI discovery and A05 validation sample, tau-PET-informed personalized cognitive composites required the lowest number of participants to detect intervention effects for all intervention strengths, although effects were stronger in ADNI (Table 2). Although no significant differences were found between the unweighted and the tau-PET-informed personalized cognitive composite in A05, our finding that the personalized cognitive composite required the lowest sample sizes also in the A05 cohort suggests that personalized cognitive composites assess cognitive change over time more sensitively than cognitive composites that are agnostic for patient-specific tau-dependent cognitive trajectories. When repeating the analysis without APOE4 as covariate, the results remained consistent, showing the lowest required sample sizes when applying the personalized cognitive composite for both cohorts (Additional file 1: Table 11).

**Table 2:** Sample size estimation to detect simulated intervention effects of 20%, 30%, and 40% in AD-spectrum patients. The model for the tau-PET-informed personalized cognitive composites was controlled for age, sex, clinical status, and APOE4 status

Required n per arm to detect an intervention effect			
Intervention effect	Episodic memory	Standard global cognitive composite	Personalized global cognitive composite
**ADNI (*N*** **= 140)**			
20%	461	168	123
30%	198	69	51
40%	109	36	27
**A05 (*N*** **= 65)**			
20%	261	279	239
30%	114	115	98
40%	63	60	52

## Discussion

Our first finding was that mapping baseline tau-PET to meta-analytical task-fMRI brain activation maps of MEM/LAN/EF/VS facilitates personalized prediction of tau-related cognitive trajectories in AD. Specifically, we found tau-PET in cognitive-domain-specific brain regions to predict domain-specific cognitive decline better than conventional tau-PET metrics (i.e., global/temporal-lobe tau-PET) which have been previously shown to be prognostic markers in AD [9, 10]. Second, we showed that using cognitive-domain-specific tau-PET as an information source to determine patient-tailored cognitive composites improves the sensitivity to assess AD-related cognitive decline compared to conventional cognitive composites. In exploratory simulated interventions, we showed further that using tau-PET-informed personalized composites as cognitive endpoints may reduce sample sizes required to detect tau-targeting treatment effects compared to conventional endpoints (e.g., MEM, global composites). Together, our independently validated findings suggest that combining tau-PET with fMRI-based mapping of cognitive abilities can facilitate the prediction of AD-related cognitive trajectories, which may improve patient-specific assessments of cognitive changes in clinical trials that target tau pathology.

First, we reported that applying task-fMRI maps of MEM/LAN/EF/VS to tau-PET in AD patients improves the prediction of cognitive-domain-specific decline compared to conventional prognostic tau-PET readouts [9, 10]. We specifically focused on these cognitive domains since they are typically assessed in cognitive test protocols for evaluating AD in standard clinical settings and clinical trials [30, 38, 39]. Our findings critically extend previous cross-sectional AD studies, emphasizing a close link between spatially heterogeneous patterns of tau deposition and neurodegeneration with heterogeneous symptom manifestation [14, 15, 40]. Since tau pathology has been shown to disrupt neuronal connectivity in preclinical [41] and clinical studies [42–45], neurotoxic tau may drive network dysfunction [46] and impairment in the cognitive domain that is supported by the tau-affected network. Supporting this, we found that higher tau-PET in brain regions that support a given cognitive domain [17, 18] is associated with faster decline in that cognitive domain. While the current study investigated tau-PET as a predictor of cognitive decline for a selected set of cognitive domains typically affected in AD [47] our proposed methodological framework can be applied for mapping tau-PET to other cognitive or non-cognitive domains, which may motivate future efforts to investigate tau-PET for predicting patient-centered disease trajectories in AD.

Second, we show that combining baseline tau-PET with task-fMRI maps of MEM/LAN/EF/VS allows determining personalized cognitive composites with increased sensitivity to detect patient-specific cognitive decline compared to conventional patient-agnostic cognitive composites. Specifically, we used baseline tau-PET signal in cognitive-domain-specific brain regions to forecast domain-specific cognitive decline. To compute a personalized composite, the actual cognitive change rates with faster tau-PET-predicted decline were weighted higher than actual change rates with slower tau-PET-predicted cognitive decline. Such patient-specific cognitive composites take into account inter-individual variability to facilitate longitudinal assessment of heterogeneous cognitive trajectories [47–49] and may thus be applied as personalized endpoints in clinical trials [7, 19]. Supporting this, we exploratorily performed simulated trials in which tau-PET-informed personalized cognitive composites increased the sensitivity to detect treatment effects compared to conventional composites, which have been previously used as endpoints in clinical trials [8, 50]. However, it should be noted that effects on estimated sample sizes for simulated intervention effects were smaller in A05 than in ADNI, potentially due to differences in cognitive tests used to obtain cognitive composites, differences in baseline scores, differences in the overall clinical characteristics between datasets, differences in frequency of amnestic versus non-amnestic phenotypes, APOE4 frequencies, or different data processing strategies. Despite small effect sizes, results were consistent across both samples; hence, our approach for assessing personalized composites may be a starting point for improving the assessment of heterogeneous cognitive trajectories to reduce sample sizes in clinical trials. Nevertheless, it will be important to replicate our findings across larger studies with harmonized data assessment and clinical characteristics.

A clear strength of the present study is the independent validation across two cohorts with different cognitive assessment protocols. Although sample sizes were smaller in the A05 cohort and effects less strong than in ADNI, the congruent results between both cohorts highlighted the robustness of our findings. Nevertheless, several limitations should be highlighted. First, flortaucipir shows off-target binding within hippocampal and subcortical areas [22]. Although these regions were a priori excluded, and Gaussian-mixture modeling was performed to further eliminate off-target binding [24, 25], our results warrant further replication with second-generation tau-PET with an improved off-target binding profile. Second, brain regions like the hippocampus are critical for cognitive function (e.g., memory); hence, second-generation tau-PET which allows hippocampal assessments [51] may further improve the prediction of cognitive decline beyond what is currently possible with flortaucipir. Third, all analyses were determined across the entire AD spectrum. Here, it will be critical to investigate the predictive accuracy of tau-PET for cognitive decline across different clinical groups once larger datasets become available. This will be particularly important for assessing the potential of tau-PET for predicting cognitive decline in patients with early AD. Fourth, the included samples show differences in the level of clinical impairment, i.e., the A05 shows lower performance on MMSE than the ADNI sample, which may confound the tau-PET-based prediction when applying the ADNI-trained models to A05 data. Here, it will be an important next step to train and validate our models across datasets which are fully comparable across all measures.

## Conclusion

In conclusion, we demonstrate independently validated evidence for tau-PET combined with fMRI-based mapping of cognitive functions as a promising tool for individualized prediction of cognitive decline in AD. Tau-PET-informed cognitive composites may facilitate detecting intervention effects and thus reduce required sample sizes in clinical trials in AD, which also may be retrospectively assessed in clinical trial data with available tau-PET.

## Supplementary Information

**Additional file 1. **Additional methods and results.

## Data Availability

All data used in this manuscript are publicly available from the ADNI database (adni.loni.usc.edu) upon registration and compliance with the data use agreement. The data that support the findings of this study are available on reasonable request from the corresponding author.

## Abbreviations

Aβ: Amyloid-beta
ADNI: Alzheimer’s Disease Neuroimaging Initiative
EF: Executive functioning composite score
LAN: Language composite score
MEM: Episodic memory composite score
VS: Visuospatial composite score
MMSE: Mini-Mental State Examination
A05: AVID-1451-A05 validation cohort
ROI: Region of interest
SUVR: Standardized uptake value ratio
