# Supplementary material for: Combining tau-PET and fMRI meta-analyses for patient-centered prediction of cognitive decline in Alzheimer’s disease
Source: Alzheimers Res Ther. 2022 Nov 7;14:166. doi: 10.1186/s13195-022-01105-5 (PMC9639286; doi:10.1186/s13195-022-01105-5)
Supplement: Supplementary file 1 — Additional file 1. Additional methods and results. [file 13195_2022_1105_MOESM1_ESM.docx]

**ADDITIONAL**

***Methods -* *Neuroimaging acquisition and preprocessing***
In ADNI T1-weighted structural MRI was recorded using standardized scanning protocols (MPRAGE; <http://adni.loni.usc.edu/methods/mri-tool/mri-analysis/>) on 3T scanners. Amyloid-PET was recorded in 4x5min frames 50-70min after ^18^F-florbetapir injection or 90-110min after ^18^F-florbetaben injection.^1^ Tau-PET was acquired 75-105min after injection of ^18^F-flortaucipir in 6x5min frames. All time frames were motion corrected and averaged to obtain mean images (see <http://adni.loni.usc.edu/methods/pet-analysis-method/pet-analysis/>). Structural MRI images were normalized to the Montreal Neurological Institute (MNI) space using the Advanced Normalization Tools (ANTs)^2^ high-dimensional warping algorithm. Amyloid-PET and tau-PET images were co-registered to native-space T1 images and normalized to MNI space using ANTs-derived normalization parameters. Amyloid-PET SUVRs were intensity normalized to the whole cerebellum and Aβ-status was determined on pre-established SUVR thresholds (Aβ+=^18^F-florbetapir SUVR>1.11^3^ or ^18^F-florbetaben SUVR>1.08). Tau-PET SUVRs were calculated using eroded white matter as a reference region.

In A05, amyloid-PET was acquired approx. 50min after ^18^F-florbetapir injection in 2x5min frames and Aβ-status (–/+) was determined by Avid investigators via expert visual read. Tau-PET was assessed approx. 80min after ^18^F-flortaucipir injection in 4x5min frames. Tau-PET images were preprocessed by Avid investigators. Specifically, native-space tau-PET images were rigidly co-registered to T1-weighted structural MRI and spatially normalized to standard MNI space using FSL ‘fnirt’ (https://fsl.fmrib.ox.ac.uk/fsl/fslwiki/FNIRT). SUVRs were obtained by intensity normalization to the eroded white matter.

## *Methods – Cognitive composites for the A05 cohort*

To calculate cognitive composites for episodic memory, language, executive functioning, and visuospatial abilities in the A05 cohort, available cognitive tests which have been previously included in cognitive composites (<https://adni.bitbucket.io/reference/docs/UWNPSYCHSUM/adni_uwnpsychsum_doc_20200326.pdf>)^4, 5^ were selected from A05 data and allocated to the respective cognitive domain (**Additional Table 1**; for descriptions of the single tests, see page 34/35 <https://clinicaltrials.gov/ProvidedDocs/60/NCT02016560/Prot_000.pdf>). For each participant, test scores within a specific cognitive domain were divided by the highest possible score of the respective test. Subsequently, the resulting values of all included tests within the cognitive domain were averaged to obtain the cognitive composite. Since there is no word limitation in the categorical word fluency test, scores were divided by the group mean plus 2 SD (instead of the highest possible score).

**Additional Table 1**. Included cognitive tests for building composite scores of episodic memory, language, executive functioning, and visuospatial abilities in the A05 validation cohort.

| **Episodic memory composite** | **Language composite** | **Executive functioning composite** | **Visuospatial composite** |
| --- | --- | --- | --- |
| Immediate and delayed recall of the Wechsler logical memory test | Categorical word fluency - animals | Digit symbol substitution test | Copying condition of the clock drawing test |
|  | Boston naming test | Digit span backward | Judgement of line orientation test |
|  |  | Trail making A and B |  |
|  |  | Categorical word fluency - animals |  |
|  |  | Copying and command condition of the clock drawing test |  |

**Additional Table 2.** Mean coefficients of the bootstrapped regression analyses for the relationship between tau and cognitive decline. The table displays average T-values, p-values, and partial R^2^ values with 95% confidence intervals (CI) for the different tau-PET ROIs (global, temporal, cognitive-domain-specific) for the AD-spectrum groups of the ADNI and A05 cohort. The models were controlled for age, sex, [in ADNI: education], clinical status, baseline score of the respective cognitive test, and APOE4 status.

|  | **Global tau** | | | **Temporal tau** | | | **Cognitive-domain-specific tau** | | | |
| --- | --- | --- | --- | --- | --- | --- | --- | --- | --- | --- |
|  | T | p | Partial R^2^ (CI) | T | p | Partial R^2^ (CI) | T | p | Partial R^2^ (CI) |  |
|  | **ADNI (N=140)** | | | | | | | | | |
| ADNI-MEM | -3.850 | 0.018 | 0.108 (0.104-0.111) | -4.766 | 0.004 | 0.152 (0.148-0.156) | -5.216 | 0.001 | 0.175 (0.171-0.179) |  |
| ADNI-LAN | -5.019 | <0.001 | 0.164 (0.160-0.167) | -4.482 | 0.002 | 0.136 (0.133-0.139) | -5.716 | <0.001 | 0.201 (0.197-0.204) |  |
| ADNI-EF | -3.454 | 0.012 | 0.088 (0.085-0.090) | -1.782 | 0.174 | 0.029 (0.027-0.030) | -4.396 | 0.002 | 0.132 (0.128-0.135) |  |
| ADNI-VS | -3.651 | 0.012 | 0.097 (0.094-0.100) | -2.988 | 0.040 | 0.070 (0.067-0.072) | -5.538 | <0.001 | 0.192 (0.188-0.192) |  |
|  | **A05 (N=65)** | | | | | | | | | |
| A05-MEM | -3.937 | 0.009 | 0.216 (0.210-0.222) | -3.730 | 0.012 | 0.199 (0.194-0.205) | -4.455 | 0.004 | 0.258 (0.252-0.264) |  |
| A05-LAN | -2.917 | 0.027 | 0.134 (0.130-0.138) | -2.848 | 0.045 | 0.131 (0.127-0.136) | -3.321 | 0.016 | 0.166 (0.161-0.170) |  |
| A05-EF | -3.863 | 0.008 | 0.210 (0.204-0.215) | -3.061 | 0.040 | 0.148 (0.143-0.154) | -4.056 | 0.005 | 0.225 (0.220-0.231) |  |
| A05-VS | -2.217 | 0.103 | 0.088 (0.084-0.092) | -1.223 | 0.332 | 0.042 (0.039-0.045) | -3.239 | 0.022 | 0.160 (0.155-0.165) |  |

**Additional Table 3.** Effect sizes between tau-PET ROIs (global, temporal, cognitive-domain-specific) and cognitive decline using paired t-test and Cohen’s d for the AD-spectrum groups of the ADNI and A05 cohort. The models were controlled for age, sex, [in ADNI: education], clinical status, baseline score of the respective cognitive test, and APOE4 status.

|  | **Global tau vs. temporal tau** | | | | **Global tau vs. cognitive-domain- specific tau** | | | | **Temporal tau vs. cognitive-domain-specific tau** | | |
| --- | --- | --- | --- | --- | --- | --- | --- | --- | --- | --- | --- |
| **AD-spectrum** | | T | p | d | | T | p | d | T | p | d |
| **ADNI (N=140)** | | | | | | | | | | | |
| ADNI-MEM | | -53.532 | <0.001 | -0.646 | | -52.869 | <0.001 | -0.978 | -18.087 | <0.001 | -0.327 |
| ADNI-LAN | | 24.707 | <0.001 | 0.479 | | -23.998 | <0.001 | -0.650 | -40.473 | <0.001 | -1.189 |
| ADNI-EF | | 67.969 | <0.001 | 1.288 | | -38.045 | <0.001 | -0.904 | -74.466 | <0.001 | -2.259 |
| ADNI-VS | | 30.133 | <0.001 | 0.582 | | -64.871 | <0.001 | -1.463 | -68.256 | <0.001 | -1.977 |
| **A05 (N=65)** | | | | | | | | | | | |
| A05-MEM | | 14.791 | <0.001 | 0.184 | | -36.792 | <0.001 | -0.447 | -48.549 | <0.001 | -0.624 |
| A05-LAN | | 2.733 | 0.006 | 0.043 | | -26.113 | <0.001 | -0.455 | -21.178 | <0.001 | -0.478 |
| A05-EF | | 46.097 | <0.001 | 0.691 | | -12.249 | <0.001 | -0.176 | -36.427 | <0.001 | -0.868 |
| A05-VS | | 50.499 | <0.001 | 0.754 | | -68.943 | <0.001 | -0.909 | -72.075 | <0.001 | -1.596 |
| **Whole sample** | | T | p | d | | T | p | d | T | p | d |
| **ADNI (N=261)** | | | | | | | | | | | |
| ADNI-MEM | | -72.526 | <0.001 | -1.013 | | -67.948 | <0.001 | -1.412 | -15.435 | <0.001 | -0.310 |
| ADNI-LAN | | 8.186 | <0.001 | 0.149 | | -29.048 | <0.001 | -0.741 | -31.915 | <0.001 | -0.900 |
| ADNI-EF | | 27.776 | <0.001 | 0.512 | | -30.018 | <0.001 | -0.787 | -41.557 | <0.001 | -1.273 |
| ADNI-VS | | 32.504 | <0.001 | 0.645 | | -68.963 | <0.001 | -1.357 | -70.623 | <0.001 | -1.871 |
| **A05 (N=111)** | | | | | | | | | | | |
| A05-MEM | | -17.324 | <0.001 | -0.174 | | -43.364 | <0.001 | -0.574 | -36.247 | <0.001 | -0.434 |
| A05-LAN | | -22.289 | <0.001 | -0.313 | | 2.548 | 0.011 | 0.038 | 17.102 | <0.001 | 0.359 |
| A05-EF | | 44.333 | <0.001 | 0.527 | | -15.363 | <0.001 | -0.209 | -36.005 | <0.001 | -0.727 |
| A05-VS | | 64.766 | <0.001 | 0.915 | | -83.400 | <0.001 | -1.240 | -91.229 | <0.001 | -1.997 |

**Additional Table 4.** Mean coefficients of the bootstrapped regression analyses for the relationship between tau SUVRs and cognitive change. The table displays average T-values, p-values, and partial R^2^ values with 95% confidence intervals (CI) for the cognitive-domain-specific ROI using SUVRs instead of gaussian-mixture modeling transformed tau positivity probabilities for the AD-spectrum groups of the ADNI and A05 cohort. The models were controlled for age, sex, [in ADNI: education], clinical status, baseline score of the respective cognitive test, and APOE4 status.

|  | **Cognitive-domain-specific tau** | | | |
| --- | --- | --- | --- | --- |
|  | T | p | Partial R^2^ (CI) |  |
|  | **ADNI (N=140)** | | |  |
| ADNI-MEM | -4.685 | 0.003 | 0.147 (0.143-0.151) |  |
| ADNI-LAN | -4.935 | <0.001 | 0.159 (0.155-0.162) |  |
| ADNI-EF | -4.539 | 0.002 | 0.139 (0.136-0.142) |  |
| ADNI-VS | -4.884 | 0.003 | 0.157 (0.153-0.161) |  |
|  | **A05 (N=65)** | | |  |
| A05-MEM | -4.320 | 0.006 | 0.247 (0.241-0.253) |  |
| A05-LAN | -2.906 | 0.029 | 0.134 (0.130-0.138) |  |
| A05-EF | -4.280 | 0.003 | 0.244 (0.238-0.249) |  |
| A05-VS | -3.178 | 0.021 | 0.155 (0.150-0.160) |  |

**Additional Table 5.** Effect sizes between tau-PET ROIs (global, temporal, cognitive-domain-specific) and cognitive decline using paired t-test and Cohen’s d. For the cognitive-domain-specific ROI, SUVRs instead of tau positivity probabilities were used. The models were controlled for age, sex, [in ADNI: education], clinical status, baseline score of the respective cognitive test, and APOE4 status.

|  | **Global tau vs. temporal tau** | | | | **Global tau vs. cognitive-domain- specific tau** | | | | **Temporal tau vs. cognitive-domain-specific tau** | | |
| --- | --- | --- | --- | --- | --- | --- | --- | --- | --- | --- | --- |
| **AD-spectrum** | | T | p | d | | T | p | d | T | p | d |
| **ADNI (N=140)** | | | | | | | | | | | |
| ADNI-MEM | | -53.532 | <0.001 | -0.646 | | -31.123 | <0.001 | -0.636 | 3.207 | 0.001 | 0.070 |
| ADNI-LAN | | 24.707 | <0.001 | 0.479 | | 3.241 | 0.001 | 0.091 | -14.421 | <0.001 | -0.427 |
| ADNI-EF | | 67.969 | <0.001 | 1.288 | | -37.656 | <0.001 | -1.037 | -74.829 | <0.001 | -2.387 |
| ADNI-VS | | 30.133 | <0.001 | 0.582 | | -41.550 | <0.001 | -1.020 | -49.672 | <0.001 | -1.532 |
| **A05 (N=65)** | | | | | | | | | | | |
| A05-MEM | | 14.791 | <0.001 | 0.184 | | -45.687 | <0.001 | -0.327 | -46.945 | <0.001 | -0.500 |
| A05-LAN | | 2.733 | 0.006 | 0.043 | | 0.565 | 0.573 | 0.008 | -1.700 | 0.089 | -0.036 |
| A05-EF | | 46.097 | <0.001 | 0.691 | | -30.631 | <0.001 | -0.380 | -48.022 | <0.001 | -1.074 |
| A05-VS | | 50.499 | <0.001 | 0.754 | | -75.937 | <0.001 | -0.869 | -75.332 | <0.001 | -1.570 |

**Additional Table 6**. Subjects characteristics for participants of in the analysis without APOE4 status inclusion.

| **ADNI (N = 272)** | **CN Aβ-**  **(n = 123)** | **CN Aβ+ (n = 64)** | **MCI Aβ+  (n = 57)** | **Dementia Aβ+  (n = 28)** | **p-value** |
| --- | --- | --- | --- | --- | --- |
| Age in years | 73.43 (7.16) | 75.64 (7.10) | 75.88 (7.06) | 76.94 (9.88) | 0.039 |
| Sex (male/female) | 52/71 | 28/36 | 32/25 | 15/13 | 0.288 |
| Years of education | 16.65 (2.47) | 16.81 (2.27) | 15.91 (2.73) | 15.89 (2.51) | 0.104 |
| MEM change rate | 0.045 (0.063)^c,d^ | 0.026 (0.070^)c,d^ | -0.087 (0.076)_a,d_ | -0.180 (0.077)^a,c^ | <0.001 |
| LAN change rate | -0.044 (0.028)^c,d^ | -0.047 (0.030)^c,d^ | -0.083 (0.038)_a,d_ | -0.135 (0.054)^a,c^ | <0.001 |
| EF change rate | -0.066 (0.035)^b,c,d^ | -0.086 (0.034)^a,d^ | -0.094 (0.030)^a^ | -0.112 (0.027)^a,b^ | <0.001 |
| VS change rate | -0.004 (0.007)^c,d^ | -0.006 (0.008)^d^ | -0.009 (0.010)^a,d^ | -0.021 (0.014)^a,b,c^ | <0.001 |
| Global tau-PET SUVR | 1.06 (0.07)^c,d^ | 1.11 (0.11)^c,d^ | 1.21 (0.22)^a,b,d^ | 1.42 (0.44)^a,b,c^ | <0.001 |
| Temporal-lobe tau-PET SUVR | 1.13 (0.09)^c,d^ | 1.22 (0.15)^c,d^ | 1.40 (0.30)^a,b,d^ | 1.68 (0.46)^a,b,c^ | <0.001 |
| Mean cognitive follow-up in years | 2.02 (0.80)^b,c,d^ | 1.62 (0.69)^a^ | 1.47 (0.71)^a^ | 1.47 (0.68)^a^ | <0.001 |
| Mean cognitive follow-up visits | 2.33 (0.55) | 2.31 (0.53) | 2.33 (0.61) | 2.32 (0.61) | 0.998 |
| **A05 (N = 116)** | **CN Aβ-**  **(n = 49)** | **CN Aβ+**  **(n = 5)** | **MCI Aβ+**  **(n = 37)** | **Dementia Aβ+**  **(n = 25)** | **p-value** |
| Age in years | 67.76 (10.36) | 77.80 (7.01) | 72.30 (8.77) | 76.04 (9.34) | 0.002 |
| Sex (male/female) | 27/22 | 3/2 | 21/16 | 11/14 | 0.753 |
| MEM change rate | 0.023 (0.027)^c,d^ | 0.015 (0.032)^c,d^ | -0.018 (0.026)^a,b,d^ | -0.038 (0.011)^a,b,c^ | <0.001 |
| LAN change rate | -0.0007 (0.030)^c,d^ | 0.009 (0.018)^d^ | -0.030 (0.030)^a,d^ | -0.070 (0.049)^a,b,c^ | <0.001 |
| EF change rate | 0.007 (0.026)^c,d^ | 0.009 (0.010)^c,d^ | -0.040 (0.040)^a,b,d^ | -0.077 (0.048)^a,b,c^ | <0.001 |
| VS change rate | 0.009 (0.030)^c,d^ | 0.0007 (0.021)^d^ | -0.032 (0.053)^a,d^ | -0.107 (0.097)^a,b,c^ | <0.001 |
| Global tau-PET SUVR | 1.01 (0.82)^c,d^ | 1.00 (0.09)^d^ | 1.21 (0.28)^a^ | 1.36 (0.32)^a,b^ | <0.001 |
| Temporal-lobe tau-PET SUVR | 1.08 (0.10)^c,d^ | 1.08 (0.09)^c,d^ | 1.37 (0.29)^a,b,d^ | 1.52 (0.31)^a,b,c^ | <0.001 |
| Mean cognitive follow-up in years | 1.47 (0.15)^c^ | 1.5 (0) | 1.32 (0.33)^a^ | 1.38 (0.28) | 0.034 |
| Mean cognitive follow-up visits | 2.96 (0.20)^c^ | 3 (0) | 2.76 (0.43)^a^ | 2.84 (0.37) | 0.034 |
| In ADNI CN: MMSE≥24, CDR=0, non-depressed; MCI: MMSE≥24, CDR=0.5, objective memory-impairment on education-adjusted Wechsler Memory Scale II, preserved activities of daily living; demented: MMSE=20-26, CDR>0.5, NINCDS/ADRDA criteria for probable AD  In A05 CN: MMSE≥29, no history of cognitive impairment; MCI: 24≤MMSE<29, showing MCI according to NIA-AA working group’s diagnostic guidelines; demented: 10<MMSE<24, showing possible or probable AD based on NIA-AA working group’s diagnostic guidelines.  Values are presented as mean (SD); p-values were derived from ANOVAs for continuous measures and from Chi-squared tests for categorical measures  MEM = episodic memory composite score; LAN = language composite score; EF = executive functioning composite score; VS = visuospatial composite score  Mean values significantly (p < 0.05, Post-hoc tests) different from—  a CN Aβ-  b CN Aβ+  c MCI Aβ+  d Dementia Aβ+ | | | | | |

**Additional Table 7**. Mean coefficients of the bootstrapped regression analyses for the relationship between tau and cognitive decline. The table displays average T-values, p-values, and partial R^2^ values with 95% confidence intervals (CI) for the different tau-PET ROIs (global, temporal, cognitive-domain-specific) for the AD-spectrum groups of the ADNI and A05 cohort. The models were controlled for age, sex, [in ADNI: education], clinical status, and the baseline score of the respective cognitive test.

|  | **Global tau** | | | **Temporal tau** | | | **Cognitive-domain-specific tau** | | | |
| --- | --- | --- | --- | --- | --- | --- | --- | --- | --- | --- |
|  | T | p | Partial R^2^ (CI) | T | p | Partial R^2^ (CI) | T | p | Partial R^2^ (CI) |  |
|  | **ADNI (N=149)** | | | | | | | | | |
| ADNI-MEM | -4.023 | 0.010 | 0.108 (0.105-0.111) | -4.853 | 0.004 | 0.147 (0.143-0.151) | -5.546 | <0.001 | 0.181 (0.177-0.186) |  |
| ADNI-LAN | -4.447 | 0.002 | 0.126 (0.123-0.129) | -3.917 | 0.004 | 0.101 (0.099-0.104) | -6.024 | <0.001 | 0.206 (0.202-0.209) |  |
| ADNI-EF | -2.943 | 0.038 | 0.063 (0.060-0.065) | -1.675 | 0.207 | 0.025 (0.024-0.027) | -3.841 | 0.008 | 0.099 (0.096-0.102) |  |
| ADNI-VS | -3.705 | 0.014 | 0.093 (0.090-0.096) | -2.964 | 0.041 | 0.064 (0.062-0.067) | -5.457 | <0.001 | 0.177 (0.173-0.182) |  |
|  | **A05 (N=67)** | | | | | | | | | |
| A05-MEM | -3.093 | 0.032 | 0.143 (0.139-0.148) | -3.227 | 0.028 | 0.154 (0.149-0.158) | -3.620 | 0.017 | 0.184 (0.178-0.189) |  |
| A05-LAN | -2.600 | 0.051 | 0.108 (0.104-0.111) | -2.701 | 0.057 | 0.116 (0.112-0.121) | -2.947 | 0.032 | 0.132 (0.128-0.136) |  |
| A05-EF | -3.424 | 0.018 | 0.168 (0.163-0.173) | -2.863 | 0.056 | 0.129 (0.124-0.134) | -3.473 | 0.015 | 0.171 (0.166-0.176) |  |
| A05-VS | -2.094 | 0.118 | 0.077 (0.073-0.080) | -1.165 | 0.343 | 0.036 (0.033-0.039) | -3.153 | 0.025 | 0.147 (0.143-0.152) |  |

**Additional Table 8.** Effect sizes between tau-PET ROIs (global, temporal, cognitive-domain-specific) and cognitive decline using paired t-test and Cohen’s d. The explorative analysis in the whole sample represents CN Aβ– plus the AD-spectrum group. The models were controlled for age, sex, [in ADNI: education], clinical status, and the baseline score of the respective cognitive test.

|  | **Global tau vs. temporal tau** | | | | **Global tau vs. cognitive-domain- specific tau** | | | | **Temporal tau vs. cognitive-domain-specific tau** | | |
| --- | --- | --- | --- | --- | --- | --- | --- | --- | --- | --- | --- |
| **AD-spectrum** | | T | p | d | | T | p | d | T | p | d |
| **ADNI (N=149)** | | | | | | | | | | | |
| ADNI-MEM | | -47.049 | <0.001 | -0.621 | | -58.704 | <0.001 | -1.128 | -27.55 | <0.001 | -0.518 |
| ADNI-LAN | | 26.161 | <0.001 | 0.517 | | -44.897 | <0.001 | -1.496 | -58.237 | <0.001 | -2.050 |
| ADNI-EF | | 54.025 | <0.001 | 0.991 | | -35.079 | <0.001 | -0.824 | -61.698 | <0.001 | -1.771 |
| ADNI-VS | | 30.233 | <0.001 | 0.649 | | -56.795 | <0.001 | -1.259 | -58.917 | <0.001 | -1.836 |
| **A05 (N=67)** | | | | | | | | | | | |
| A05-MEM | | -12.537 | <0.001 | -0.135 | | -37.674 | <0.001 | -0.460 | -30.095 | <0.001 | -0.343 |
| A05-LAN | | -8.065 | <0.001 | -0.127 | | -22.033 | <0.001 | -0.363 | -9.416 | <0.001 | -0.221 |
| A05-EF | | 34.341 | <0.001 | 0.472 | | -3.130 | 0.002 | -0.040 | -25.132 | <0.001 | -0.523 |
| A05-VS | | 47.001 | <0.001 | 0.736 | | -68.944 | <0.001 | -0.921 | -69.068 | <0.001 | -1.575 |
| **Whole sample** | | T | p | d | | T | p | d | T | p | d |
| **ADNI (N=272)** | | | | | | | | | | | |
| ADNI-MEM | | -76.187 | <0.001 | -1.093 | | -74.216 | <0.001 | -1.598 | -19.499 | <0.001 | -0.411 |
| ADNI-LAN | | 3.640 | <0.001 | 0.065 | | -44.548 | <0.001 | -1.433 | -44.558 | <0.001 | -1.507 |
| ADNI-EF | | 12.359 | <0.001 | 0.207 | | -25.659 | <0.001 | -0.695 | -29.861 | <0.001 | -0.898 |
| ADNI-VS | | 29.224 | <0.001 | 0.585 | | -67.528 | <0.001 | -1.452 | -68.376 | <0.001 | -1.958 |
| **A05 (N=116)** | | | | | | | | | | | |
| A05-MEM | | -25.710 | <0.001 | -0.271 | | -41.896 | <0.001 | -0.598 | -28.109 | <0.001 | -0.347 |
| A05-LAN | | -26.246 | <0.001 | -0.362 | | 3.685 | <0.001 | 0.052 | 21.564 | <0.001 | 0.427 |
| A05-EF | | 26.844 | <0.001 | 0.311 | | -9.433 | <0.001 | -0.113 | -23.865 | <0.001 | -0.425 |
| A05-VS | | 54.515 | <0.001 | 0.832 | | -79.871 | <0.001 | -1.156 | -81.257 | <0.001 | -1.867 |

**Additional Table 9.** Mean coefficients of the bootstrapped regression analyses for the relationship between tau SUVRs and cognitive change. The table displays average T-values, p-values, and partial R^2^ values with 95% confidence intervals (CI) for the cognitive-domain-specific ROI using SUVRs instead of gaussian-mixture modeling transformed tau positivity probabilities for the AD-spectrum groups of the ADNI and A05 cohort. The models were controlled for age, sex, [in ADNI: education], clinical status, and baseline score of the respective cognitive test.

|  | **Cognitive-domain-specific tau** | | | |
| --- | --- | --- | --- | --- |
|  | T | p | Partial R^2^ (CI) |  |
|  | **ADNI (N=149)** | | |  |
| ADNI-MEM | -4.943 | 0.001 | 0.151 (0.147-0.155) |  |
| ADNI-LAN | -5.256 | <0.001 | 0.166 (0.162-0.169) |  |
| ADNI-EF | -4.295 | 0.004 | 0.119 (0.116-0.122) |  |
| ADNI-VS | -4.781 | 0.003 | 0.144 (0.140-0.148) |  |
|  | **A05 (N=67)** | | |  |
| A05-MEM | -3.335 | 0.026 | 0.162 (0.157-0.167) |  |
| A05-LAN | -2.440 | 0.071 | 0.098 (0.094-0.102) |  |
| A05-EF | -3.617 | 0.011 | 0.183 (0.177-0.188) |  |
| A05-VS | -3.064 | 0.026 | 0.140 (0.136-0.145) |  |

**Additional Table 10.** Effect sizes between tau-PET ROIs (global, temporal, cognitive-domain-specific) and cognitive decline using paired t-test and Cohen’s d. For the cognitive-domain-specific ROI, SUVRs instead of tau positivity probabilities were used. The models were controlled for age, sex, [in ADNI: education], clinical status, and baseline score of the respective cognitive test.

|  | **Global tau vs. temporal tau** | | | | **Global tau vs. cognitive-domain- specific tau** | | | | **Temporal tau vs. cognitive-domain-specific tau** | | |
| --- | --- | --- | --- | --- | --- | --- | --- | --- | --- | --- | --- |
| **AD-spectrum** | | T | p | d | | T | p | d | T | p | d |
| **ADNI (N=149)** | | | | | | | | | | | |
| ADNI-MEM | | -47.049 | <0.001 | -0.621 | | -34.633 | <0.001 | -0.730 | -2.654 | 0.008 | -0.060 |
| ADNI-LAN | | 26.161 | <0.001 | 0.517 | | -23.219 | <0.001 | -0.782 | -37.662 | <0.001 | -1.336 |
| ADNI-EF | | 54.025 | <0.001 | 0.991 | | -45.394 | <0.001 | -1.244 | -70.409 | <0.001 | -2.185 |
| ADNI-VS | | 30.233 | <0.001 | 0.649 | | -34.967 | <0.001 | -0.830 | -42.96 | <0.001 | -1.393 |
| **A05 (N=67)** | | | | | | | | | | | |
| A05-MEM | | -12.537 | <0.001 | -0.135 | | -29.849 | <0.001 | -0.225 | -11.24 | <0.001 | -0.104 |
| A05-LAN | | -8.065 | <0.001 | -0.127 | | 11.394 | <0.001 | 0.159 | 12.421 | <0.001 | 0.274 |
| A05-EF | | 34.341 | <0.001 | 0.472 | | -16.187 | <0.001 | -0.171 | -33.24 | <0.001 | -0.648 |
| A05-VS | | 47.001 | <0.001 | 0.736 | | -75.508 | <0.001 | -0.849 | -70.613 | <0.001 | -1.528 |

**Additional Table 11.** Sample size estimation to detect simulated intervention effects of 20%, 30%, and 40% in AD-spectrum patients. The model for the tau-PET-informed personalized cognitive composites was controlled for age, sex, and clinical status.

| **Required n per arm to detect an intervention effect** | | | |
| --- | --- | --- | --- |
| **Intervention effect** | **Episodic memory** | **Standard global cognitive composite** | **Personalized global cognitive composite** |
| **ADNI (N=149)** |  |  |  |
| 20 % | 444 | 163 | 65 |
| 30 % | 191 | 67 | 27 |
| 40 % | 104 | 35 | 14 |
| **A05 (N=67)** |  |  |  |
| 20 % | 265 | 279 | 257 |
| 30 % | 115 | 114 | 105 |
| 40 % | 64 | 60 | 55 |


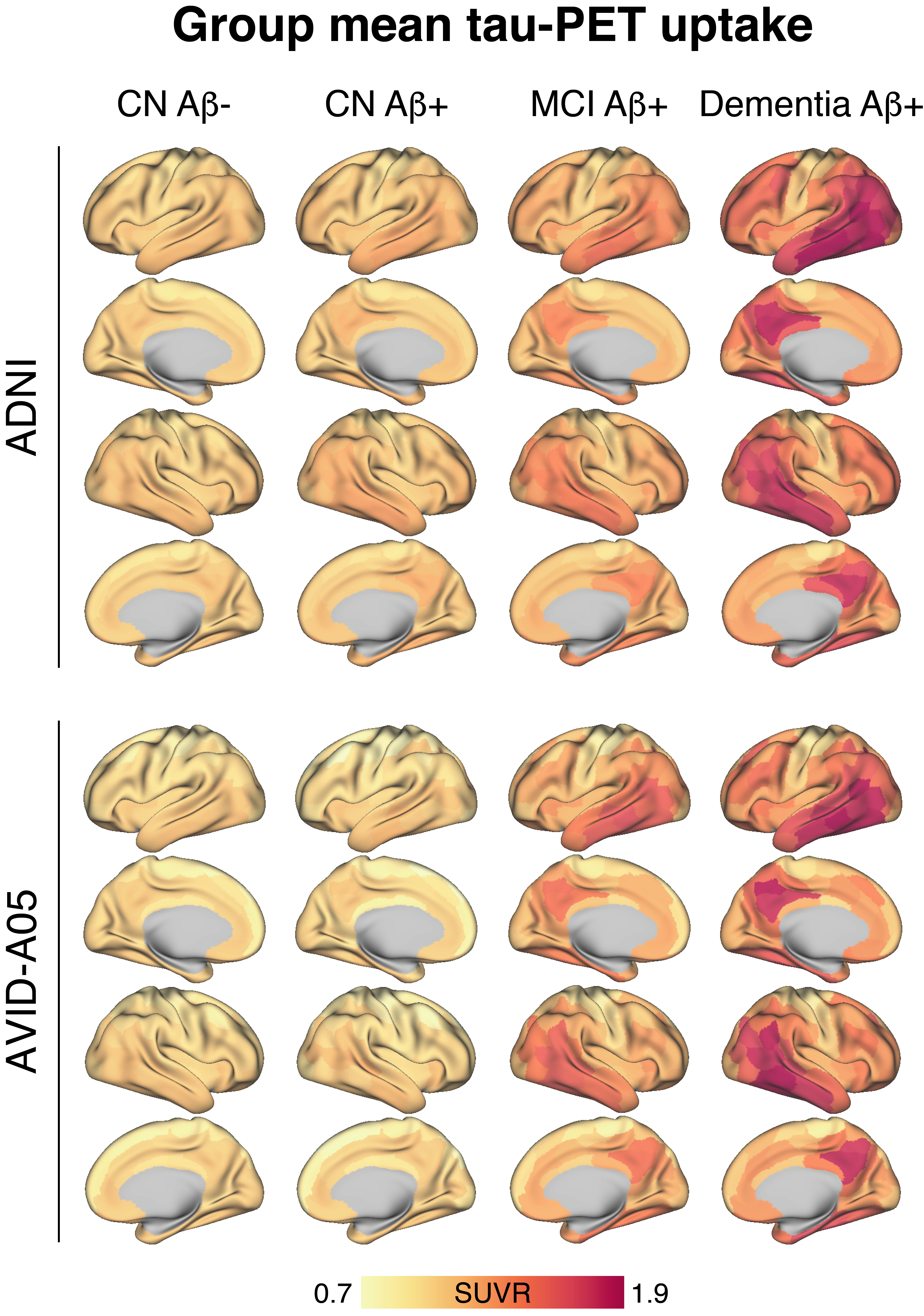


**Additional Figure 1.** Surface rendering of group mean global tau-PET SUVRs for the ADNI and A05 cohorts, stratified by diagnostic group. Surface renderings have been parcellated using 200 neocortical ROIs from the Schaefer brain atlas.^6^


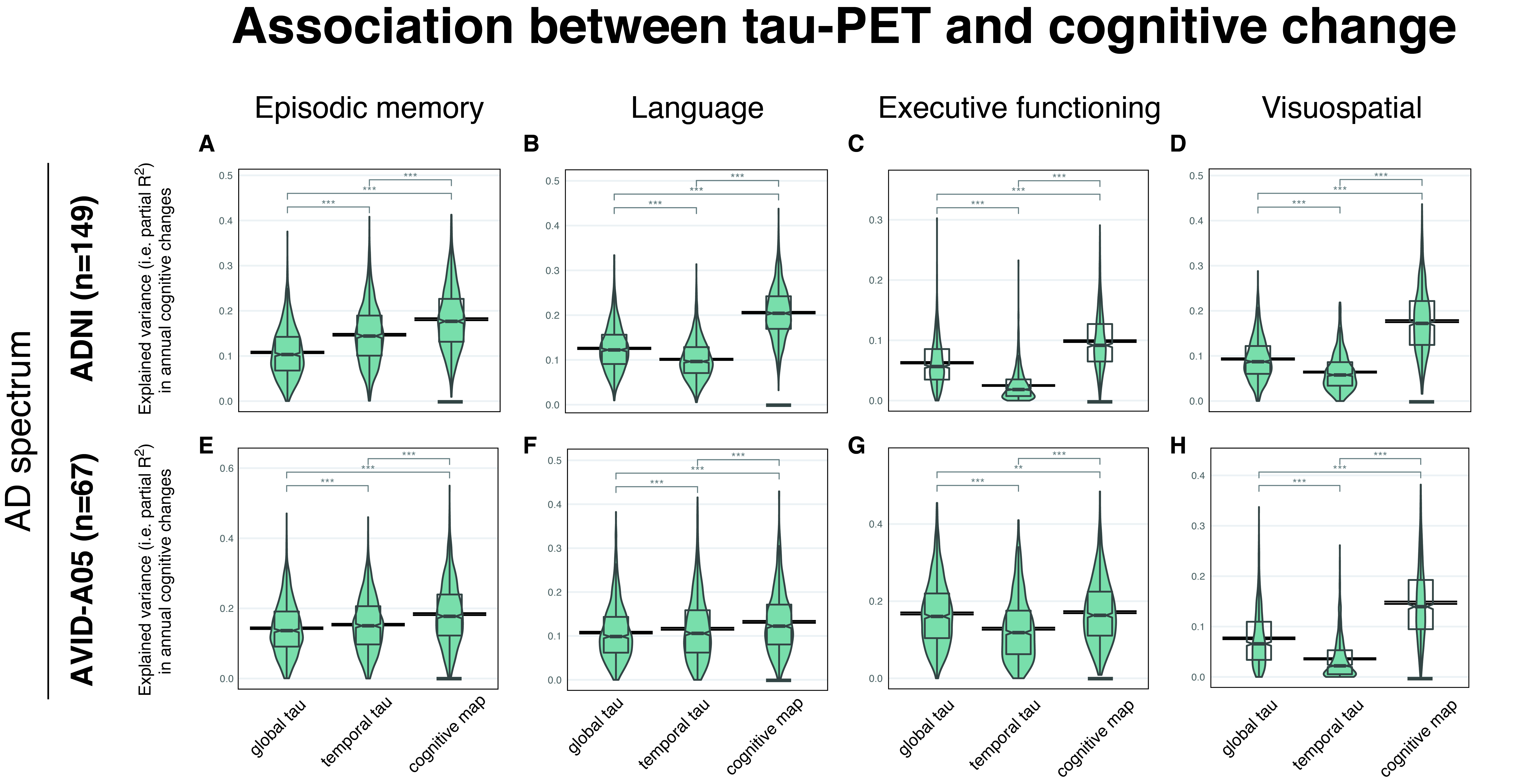


**Additional Figure 2.** Bootstrapped linear models (1000 iterations) revealed that partial R^2^ values (explained variance in annual cognitive changes) were higher for cognitive-domain-specific tau than for global or temporal tau-PET. The boxplots illustrate the partial R^2^ distributions for episodic memory, language, executive functioning, and visuospatial abilities within the AD-spectrum (ADNI cohort: *A-D*; A05 cohort: *E-H*). The models were controlled for age, sex, [in ADNI: education], clinical status, and the baseline score of the respective cognitive test. R^2^-distributions across different regions of interests (global vs. temporal vs. cognitive-domain-specific tau-PET) were compared with paired t-tests. 95% confidence intervals for the mean are displayed as horizontal lines. Within each panel, the tau-PET ROI with the highest explained variance is highlighted by an underscore. *= p<0.05, **= p<0.01, ***=p<0.001.

**
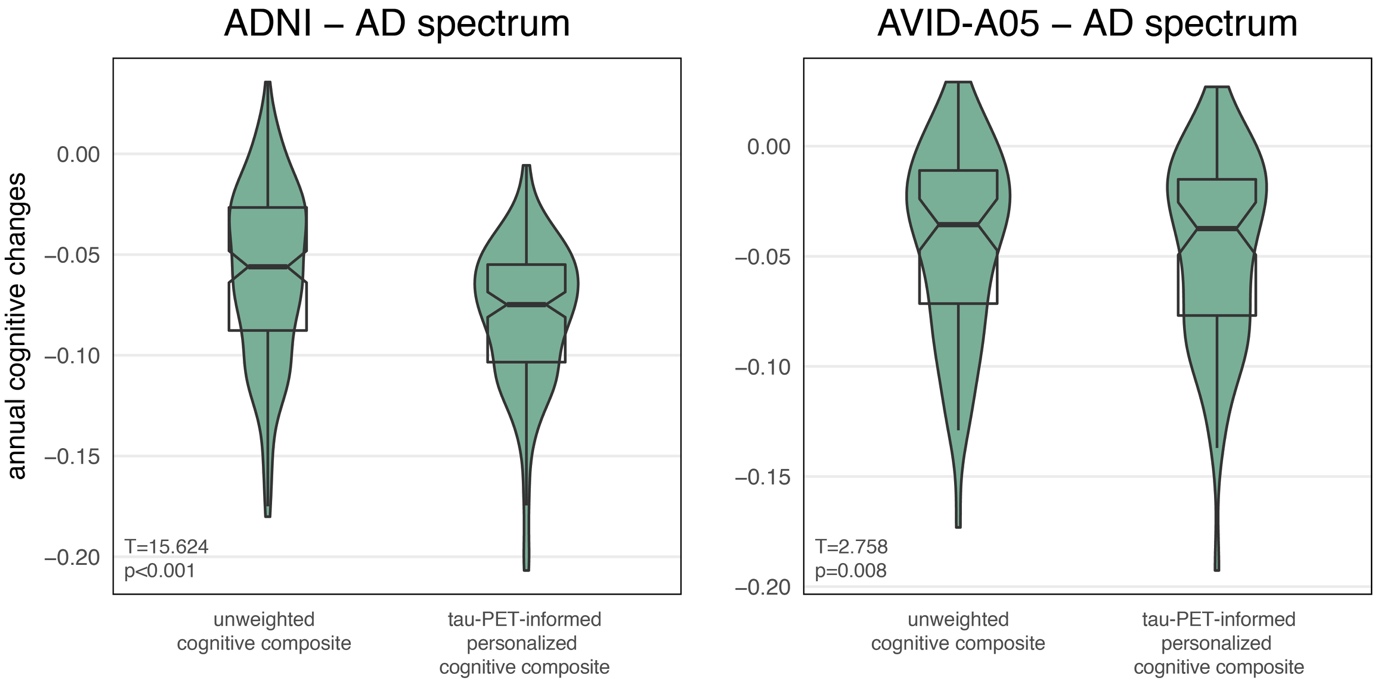
**

**Additional Figure 3.** Comparison of unweighted (i.e. average of MEM/LAN/EF/VS) and tau-PET-informed personalized cognitive composites for AD-spectrum patients (i.e. Aβ+) within the ADNI cohort *(A)* and the A05 cohort *(B)*. Statistics were derived from paired t-tests. The model for the tau-PET-informed personalized cognitive composites was controlled for age, sex, and clinical status.

**REFERENCES**

1. Landau SM, Mintun MA, Joshi AD, Koeppe RA, Petersen RC, Aisen PS *et al.* Amyloid Deposition, Hypometabolism, and Longitudinal Cognitive Decline. *Annals of neurology* 2012; **72**(4)**:** 578-586.

2. Avants BB, Tustison NJ, Song G, Cook PA, Klein A, Gee JC. A reproducible evaluation of ANTs similarity metric performance in brain image registration. *Neuroimage* 2011; **54**(3)**:** 2033-2044.

3. Landau SM, Mintun MA, Joshi AD, Koeppe RA, Petersen RC, Aisen PS *et al.* Amyloid deposition, hypometabolism, and longitudinal cognitive decline. *Ann Neurol* 2012; **72**(4)**:** 578-586.

4. Malek-Ahmadi M, Chen K, Perez SE, He A, Mufson EJ. Cognitive composite score association with Alzheimer's disease plaque and tangle pathology. *Alzheimers Res Ther* 2018; **10**(1)**:** 90.

5. Langbaum JB, Hendrix SB, Ayutyanont N, Chen K, Fleisher AS, Shah RC *et al.* An empirically derived composite cognitive test score with improved power to track and evaluate treatments for preclinical Alzheimer's disease. *Alzheimers Dement* 2014; **10**(6)**:** 666-674.

6. Schaefer A, Kong R, Gordon EM, Laumann TO, Zuo XN, Holmes AJ *et al.* Local-Global Parcellation of the Human Cerebral Cortex from Intrinsic Functional Connectivity MRI. *Cereb Cortex* 2018; **28**(9)**:** 3095-3114.
